# John M. Woodworth, M. D.

**Published:** 1879-06

**Authors:** 


### JOHN MAYNARD WOODWORTH.

IN MEMORIAM.—The death of Surgeon General Woodworth has caused a profound feeling of sorrow wherever he was known, and especially in Government official circles. Notice of his death was officially promulgated by the Treasury Department as follows:

TREASURY DEPARTMENT, Washington, March 14, 1879.

*To the Medical Officers of the U. S. Marine-Hospital Service:*

It is my painful duty to announce to the Medical Officers of the Marine Hospital Service, the death of Surgeon-General John M. Woodworth, which occurred in this city March 14th, 1879.

Surgeon-General Woodworth was born in Chemung Co., New York, August 15th, 1837. He entered the service of the United States as an Acting Assistant Surgeon of the Army in 1862, was soon after appointed Assistant Surgeon of Volunteers, and in 1863 promoted to Surgeon, and afterward to Medical Inspector and Medical Director of the Army of the Tennessee. He was previous to his leaving the Army brevetted Lieutenant-Colonel. His connection with the Marine-Hospital Service dates from its reorganization in 1871, and the history of the Service since that date is mainly identified with his own, for the work of reorganization has been solely intrusted to him since its commencement. As a mark of respect to the memory of so distinguished an officer, whose fame as a sanitarian was not only national but world-wide, the flags of all U. S. Marine Hospitals, will be displayed at half-mast on the day following the receipt of this order. Respectfully, (Signed) JOHN SHERMAN,

Secretary of the Treasury.

Dr. John M. Woodworth was born in Chemung Co., N. Y., Aug. 15th, 1837, and died March 14th, 1879. The year following his parents came to Illinois and settled on a farm near Kaneville, Kane Co. At the age of thirteen years he entered the Christian life which thereafter ever showed its impress upon all his acts both private and official. He went to Chicago when a young man and engaged in the drug business with his brother, who was a practicing physician. Being interested in scientific subjects, he became a member of the Chicago Academy of Sciences, and did a great deal of work toward putting it on a sound basis and increasing its usefulness. To this end, and at the instance of Robert Kennicott, the founder of the Academy, he spent a year at the Smithsonian Institution at Washington with Profs. Henry and Baird, securing duplicates for the Academy. After returning he took up the study of medicine, graduating from the Chicago Medical College in the spring of 1862. Immediately following his graduation he received an appointment as Acting Assistant Surgeon at Camp Douglas, and soon thereafter was appointed Assistant Surgeon of the First Illinois Light Artillery, and assigned to batteries A and B, recruiting in Chicago. He was subsequently promoted to the rank of Surgeon, and appointed Medical Inspector of the Fifteenth Army Corps, commanded by Gen. Logan. At the close of the war he had attained the position of Medical Director of the Army of the Tennessee, with the rank of Brevet Lieutenant-Colonel in recognition of efficient service. Before resuming practice, he went to Europe with Dr. E. O. F. Roler, of Chicago, and remained abroad a year, traveling and studying. On his return he formed a partnership with the venerable Dr. Ira Hatch, of Chicago, and entered active practice. In 1867 he was chosen Professor of Comparative Anatomy by the Faculty of the University of Chicago, and afterwards was appointed Professor of Physiology at the Chicago Medical College. At the same time he held the position of Surgeon of the Soldiers' Home and Examining Surgeon of the Pension Service. These positions he continued to fill until, on the passage of the act reorganizing the Marine Hospital Service, in 1871, he received the appointment of chief of the newly-created bureau, the duties of which office called him to Washington, where he has since resided. It is in this latter position that he has done his most important work. Within the necessary limits of this sketch it would be impossible to give any adequate idea of the amount and character of the labor involved, not only in correcting the abuses which had grown up under the previous no-system of furnishing relief to sick sailors, but in carrying into effect the provisions of an act which, on its face, was crude and vague to a perplexing degree. Dr. Woodworth labored incessantly and unsparingly, both of himself and others, to remedy these defects ; and so successfully that his untimely demise cannot affect the permanency of the service he has rendered. His fitting monument is in the compact, efficient, economical organization he has left behind him which supplies medical and surgical relief to over 40,000 sailors, composing our commercial navy.

He has left his impress on hospital construction and administration ; the Marine Hospital at Lake View, a model of the best of the old style, and that at San Francisco, embodying the improvements and reform of the modern styles.

He was complimented in general orders for his energy in the establishment of field hospitals during the Atlanta campaign, and again, in the subsequent campaign, for his moving ambulance hospital, which carried one hundred wounded men from Atlanta to Savannah, and placed them in hospital there without the loss of a single life, although a number of important operations had to be performed by the way. His principal scientific works and papers are: the "Mystery of Life," published in 1871 : "Regulations of the United States Hospital Marine Service," 1873; "Cholera in 1873 in the United States ;" "Migrants and Sailors in their Relation to Public Health;" "Safety of Ships and those who travel in Them," and "Quarantine with Reference to Yellow Fever." His work on the "Nomenclature of Diseases," 1874, is the standard reference of the Marine Hospital Service.

With his work in connection with epidemic disases—notably in connection with the cholera epidemic of 1873, and the yellow fever epidemic of 1878—both the medical profession and the public are well informed. The bulletins from his office, collating the facts of the public health in almost all civilized countries, are rapidly acquiring a value similar to the weather bulletins of the Signal Service. It is hoped that a fitting successor has been found to carry to completion the efforts he has inaugurated in this and other directions.

Dr. Woodworth married Miss Maggie Hannah of Chicago, in 1873, and his family is connected with many of the oldest citizens of our city.

A little time before his death he became conscious. Then he dictated some important letters ; sent telegrams to Mobile and New Orleans in relation to the formation of Boards of Public Health ; also gave directions about a $500 check, which he had for the yellow fever fund. Vice President Wheeler asked him if he was ready to go. He said " I am ready." That Christ had died for him. Then placing his hand upon his breast said Christ was there, and to his friends farewell forever. The unconscious state soon returned, and in a few hours he sank peacefully away, as in a quiet slumber.

The funeral services of Dr. Woodworth were held in Washington on the 16th, and the body was laid away in the beautiful Rock Creek Cemetery. The funeral, which was an imposing one, was managed by the Treasury Department. Five Bureau officers—Genl. Raum, Commissioner of Internal Revenue; Mr. Burchard, Director of the Mint; Gen. Schofield, Register of the Treasury; Col. Irish, Superintendent of the Bureau of Engraving and Printing; and Judge Carter, First Comptroller of the Treasury—acted as pall-bearers.

The President, Secretary Sherman, Assistant Secretary Hawley, Senator Logan, and a great concourse of persons in private and official life attended. The services were conducted by Drs. Cuthbert, of the First Baptist Church, Paxton, of the N. Y. Avenue Presbyterian Church, Rankin, of the Congregational Church, and Rev. C. K. Marshall, of Vicksburg, Mississippi. The floral offerings, which were of the choicest flowers, were most beautiful. A large crown surmounted by a cross of immortelles and violets; a cross and anchor were sent by Mrs. Hayes. Secretary Sherman sent a cross, and the employes of Dr. Woodworth's office sent an anchor which was surrounded by a fine floral wreath.

A special meeting of the executive committee of the Yellow Fever National Relief Commission was held Saturday evening, March 15th, at Willard's Hotel to take action relative to the death of Surgeon-General Woodworth. There were present ex-Governor Shepherd (chairman), Lewis J. Davis, Simon Wolf, George Hill, jr., A. S. Solomons, John F. Cook, ex-Senator Pease, Col. McArdle, Captain Lake, and Rev. C. K. Marshall, of Mississippi, and William Dickson, secretary.

Ex-Governor Shepherd announced the object for which the meeting was called, and referred to the valuable services of the deceased as a member of the Commission.

Simon Wolf, Esq., presented the following resolutions, which were adopted:—

The Yellow Fever National Relief Commission having learned with profound regret, of the sudden death of their distinguished colleague, John M. Woodworth, and being desirous to fittingly express its sorrow, resolves that,

WHEREAS, John M. Woodworth, Surgeon-General, of the Marine Hospital Service, and a member of this Commission, has been taken from our midst, and

WHEREAS, we, the members of the Commission, desires to honor his great work, integrity, and nobility of character and devotion to duty, therefore,

*Resolved*, That in the death of John M. Woodworth science has lost an eminent disciple, humanity an earnest laborer, and the United States Government an active, indefatigable, and zealous official.

*Resolved*, That as an associate he was gentlemanly, courteous, self-sacrificing, and gave the fullest measure of his ability and influence to the success of the aims and objects of this Commission, and that he fell a soldier in the sacred cause of humanity, his large heart and brain being enlisted in the promotion of such legislation as would nationalize sanitary science, and prevent the introduction and spread of contagious diseases.

*Resolved*, That we tender to his widow our profound sympathy and respect, and that the name and memory of John M. Woodworth will ever be held in high esteem as a benefactor of his race.

*Resolved*, That this Commission attend his funeral in a body, and that a copy of these resolutions be transmitted to his bereaved family, and published in the daily papers of this city.

Colonel McArdle, of Mississippi, delivered a beautiful and eloquent tribute to the memory of the diseased, and as a representative of the section stricken by the pestilence last summer, related in detail the philanthropic and noble services rendered by Dr. Woodworth to aid his people, who would mourn in sadness the death of their stranger benefactor. The Rev. C. K. Marshall also spoke in fitting terms of the deceased.

At a meeting of the principal officers of the Treasury Department, held at the rooms of the Secretary, on the fourteenth day of March, 1879, the following named gentlemen were appointed to prepare resolutions expressive of their appreciation of the character and services of Dr. John M. Woodworth, Supervising Surgeon General of the Marine Hospital Service, whose death had been announced, and their sympathy with the family of the deceased, and to make arrangements to attend the funeral: Mr. French, Assistant Secretary; Mr. Raum, Commissioner of Internal Revenue; Mr. Porter, First Comptroller; Mr. Burchard, Director of the Mint; Mr. Johnson, Commissioner of Customs.

At an adjourned meeting, on the fifteenth day of March, 1879, said committee reported the following resolutions, which were adopted:

The death of one whose life has been filled with good deeds, often does not excite that sense of loss which is necessary to create completeness of sorrow. His good deeds so fill the memory that he seems yet to survive in them, and being dead he yet seems to live. The life of a great physician is a perpetual benefaction. He appears to live for others rather than himself. Dr. John M. Woodworth, whose death we now lament, led a strenuous life, highly devoted to duty.

Beginning as a pharmacist, dependent for a livelihood upon his own faculties, his aspiring nature instinctively sought a broader and higher field of exertion. He engaged zealously in medical studies, graduated as a doctor of medicine, and being by nature earnest and resolute, immediately started on a career of professional distinction. He sought intercourse with eminent minds, and as he was zealous to receive, so, happily, he was zealous to impart knowledge. At an early age he won attention by the productions of his pen, and was sought as a member of various learned societies.

Entering the army as an assistant post surgeon, the force of his merit raised him successively from step to step in promotion, until at the age of 27 he had risen to be medical director and inspector of the army of the Tennessee. He received a brevet in token of highly meritorious service in Sherman's great march to the sea.

When the war closed, though his learning and experience were large, his aspiration for greater knowledge could not be held in check. He visited Europe, studying in the hospitals of Berlin and Vienna, and devoting himself with unremitting assiduity, until his savings were exhausted, to exploring the wider fields of professional knowledge. It was not long after his return until he was invited to take the honorable post at which he has fallen, and which he has magnified by his learning and administrative ability, and organized to be an instrument of great and enduring usefulness. When the yellow fever broke out last summer, and ravaged so great a part of the South, his soul was touched by the manifold recitals of sufferings and sorrow, and he burned with an inextinguishable ardor to make his office an efficient means to vanquish the disease then, and to prevent its return thereafter.

He was quick, when the horrid specter of the Asiatic plague rose in the East, to begin to study of the origin and progress of that malady, and to devise methods by which its access to our own shores might be prevented, and if all precautions failed, its ravages might be mitigated and diminished. In the pursuit of these investigations, and in the organization of his office to meet the great emergencies which it might be invoked to confront, he literally allowed himself no rest. The hours of each day seemed too brief for the tasks that crowded upon it. Though he felt the sense of overwork and exhaustion, labor to him was pleasure, because duty, as he thought, invoked him to it.

His manner was modest and unobtrusive, and in domestic life singularly sweet, but his spirit was heroic. In the presence of a great duty, demanding high exertions, health and life were held of no account, success must be attained; he did not pause to think whether he might enjoy a part of its fruition. When just before his death he awoke for a moment to consciousness, he asked, "Where am I to lie?" And when told where his remains would be consigned to repose, a smile of satisfaction irradiated his features and the avenues to the world were then closed forever. No soldier in the field, dying in a good cause, ever offered up his life more disinterestedly than he has done in sacrificing his own in devising means for saving the lives of others.

With this estimate of the life and character of our departed friend, it is

*Resolved*, That we lament the death of one whose life gave promise of such extended and continuing usefulness, and we tender to his devoted wife and to his kindred our sincere sympathy.

*Resolved*, That the heads of the various bureaus of this Department will in a body attend the funeral of Dr. Woodworth, tomorrow at Le Droit Park; that the proceedings be placed on the records of this Department; and that a copy of the preamble and resolutions be transmitted to the widow of the deceased, and furnished to the daily papers for publication.

HENRY F. FRENCH, H. C. BURCHARD,
GREEN B. RAUM, W. C. JOHNSON,
ALBERT G. PORTER, Committee.

---

M. RANVIER has been lately devoting some attention to the study of the structure of the cornea, and on February 8th communicated the results to the Société de Biologie. According to him, the corpuscles of the cornea cannot be seen in the normal eye of a living animal; they only appear when the eye in question has been kept for some time in aqueous humor. If the cells of the cornea become visible under the influence of steam, this is due to imbibition by the membrane. The fibers of the cornea are very hygrometric. A bull's eye, if plunged into distilled water, will increase in diameter several times.

